# The tubulin code in acute ischemic stroke: post-translational reprogramming of the neuronal cytoskeleton and implications for phase-specific neuroprotection

**DOI:** 10.3389/fnins.2026.1971973

**Published:** 2026-09-23

**Authors:** David Bar-Or, Gregory Thomas, Alexa Schroder, Christian Burrell, Renee Groechel, Daniel Paredes

**Affiliations:** 1 Stroke Research Department, Swedish Medical Center, Englewood, CO, United States; 2 Knoebel Institute of Healthy Aging, University of Denver, Denver, CO, United States; 3 Department of Chemistry and Biochemistry, University of Denver, Denver, CO, United States; 4 Neurology Department, Swedish Medical Center, Englewood, CO, United States; 5 Department of Electrical and Computer Engineering, University of Denver, Denver, CO, United States; 6 Department of Neurology, Johns Hopkins School of Medicine, Baltimore, MD, United States

**Keywords:** acute ischemic stroke, alpha-tubulin acetylation, cytoskeleton, detyrosination, HDAC6, lactylation, microtubules, neuroprotection

## Abstract

Acute ischemic stroke initiates a rapid convergence of excitotoxicity, calcium overload, oxidative stress, neuroinflammation, and energetic failure. Although reperfusion therapies remain central to acute care, they do not directly address the structural failure of injured neuronal networks. This narrative review examines the neuronal microtubule cytoskeleton as an active regulatory platform whose function is governed by the tubulin code: tubulin isotype composition and post-translational modifications. The review integrates evidence that ischemia alters alpha-tubulin K40 acetylation, detyrosination, delta-2 tubulin accumulation, and polyglutamylation, while also examining emerging roles for palmitoylation, lactylation, and glycylation-related PTM cross-talk. Particular emphasis is placed on the HDAC6-alpha-tubulin acetylation axis, MIF-AIF parthanatos signaling, vasohibin-dependent detyrosination, CCP1-dependent control of polyglutamylation, TUBA4A-GSK3beta-Tau scaffolding, beta-tubulin isotype effects on whole-lattice behavior, and lactylation-mediated repair. Together, these pathways support a phase-specific model in which early cytoprotection preserves transport-competent microtubules, whereas later recovery requires adaptive cytoskeletal remodeling and neuroplasticity.

## Introduction

Acute ischemic stroke is a leading cause of permanent neurological disability worldwide, as the result of a devastating cascade of excitotoxicity, neuroinflammation, oxidative stress, and rapid energetic failure (Wang et al., 2016; Esmaili-Shahzade-Ali-Akbari et al., 2026). While standard interventions such as hyperacute thrombolysis and endovascular thrombectomy provide effective recanalization, their efficacy is severely constrained by narrow therapeutic windows, leaving most patients with permanent neurological deficits (Esmaili-Shahzade-Ali-Akbari et al., 2026).

Emerging research has shifted focus toward the active structural and regulatory platforms of the neuronal cytoskeleton with the goal to develop targeted neuroprotective and regenerative therapies. Microtubules, hollow cylindrical polymers assembled from α- and β-tubulin heterodimers, are not merely passive structural scaffolds (Liu et al., 2022; Cui et al., 2026; Nair et al., 2026); instead, they are active conduits for intracellular transport, master regulators of cellular morphology, and coordinators of synaptic plasticity (Praveen and Talwar, 2025; Cui et al., 2026).

The functional diversity of these structures is governed by the “tubulin code”—a sophisticated regulatory layer comprising distinct tubulin isotypes and a vast repertoire of post-translational modifications (PTMs) (Teoh and Bartolini, 2025; Cui et al., 2026; Nair et al., 2026). During cerebral ischemia, this tubulin code undergoes profound pathological reprogramming (Wang et al., 2016; Hu et al., 2022; Cui et al., 2026). Unraveling the spatial and temporal alterations in tubulin modifications, and the specific enzymes that write, read, and erase them, provides a powerful framework for developing targeted neuroprotective and regenerative therapies (Guo et al., 2025; Teoh and Bartolini, 2025). The global ischemia-to-tubulin-code framework is summarized in Figure 1.

**Figure 1:**
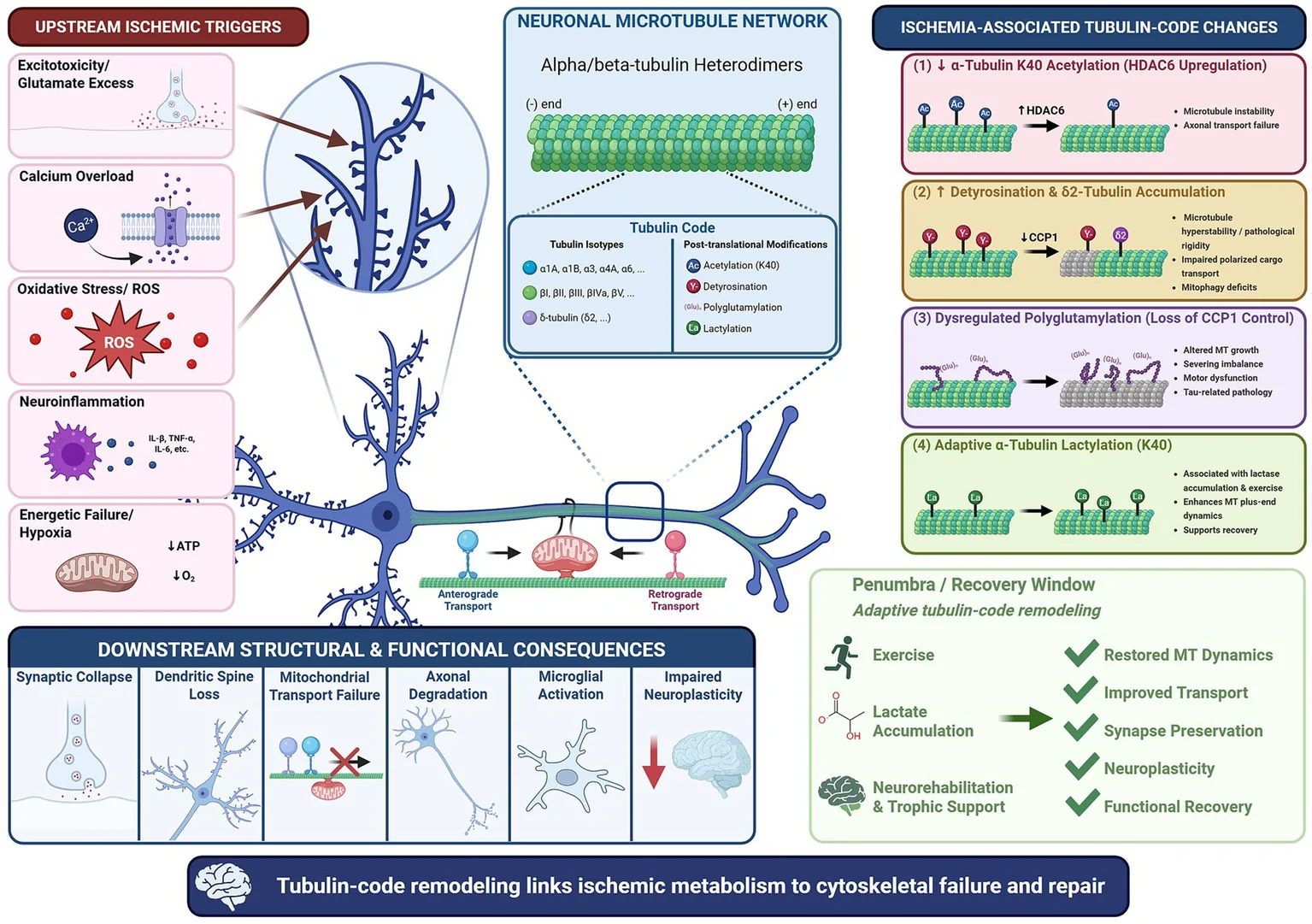
Pathophysiological reprogramming of the tubulin code in acute ischemic stroke. The schematic integrates upstream ischemic triggers with downstream changes in alpha-tubulin acetylation, detyrosination/delta-2 tubulin accumulation, polyglutamylation, and lactylation, emphasizing the transition from cytoskeletal failure to potential repair. Created in BioRender. Hausburg (2026a). https://BioRender.com/jsvdt9l.

## Structural and genetic architecture of the neuronal tubulin code

The complexity of the tubulin code in the mammalian central nervous system arises from two intersecting mechanisms: isotype diversity and a dense array of enzymatic post-translational modifications (Teoh and Bartolini, 2025; Nair et al., 2026). Humans express nine distinct α- tubulin genes and ten β-tubulin genes, which are expressed in highly specific temporal and spatial patterns (Nair et al., 2026). In adult mature neurons, the microtubule network is exceptionally heterogeneous, characterized by high expression levels of the βIII-tubulin isotype (TUBB3), which features a unique C-terminal tail that confers intrinsic resistance to oxidative stress and maintains highly dynamic microtubule populations (Nair et al., 2026).

The modern tubulin-code framework was developed substantially through work by Janke and colleagues, who emphasized that microtubule functional identity emerges from the combination of tubulin isotypes and post-translational modifications rather than from polymer mass alone (Janke and Bulinski, 2011; Magiera and Janke, 2014; Gadadhar et al., 2017). These modifications can change lattice mechanics directly or alter the recruitment of motors, microtubule-associated proteins, severing enzymes, and other effectors. This framework is especially relevant in the nervous system, where stable and dynamic microtubule domains coexist within the same cell and where disruption of PTM-writing or PTM-erasing enzymes can produce distinct neurodevelopmental and neurodegenerative phenotypes.

Other crucial neuronal isotypes, such as βII-tubulin (TUBB2A/TUBB2B), are highly enriched in adult cortical networks, where they contribute to the structural architecture necessary for synaptic transmission (Nair et al., 2026; Yang et al., 2023). Mammalian brain α- and β-tubulins exhibit approximately 40% amino acid sequence homology with each other, though across different species, individual α- or β-isotypes display extreme evolutionary conservation of approximately 88–95% similarity (Nair et al., 2026).

While the folded “tubulin body” forms the core of the microtubule polymer, the highly divergent, intrinsically disordered C-terminal tails (CTTs) project outward from the outer surface of the ordered lattice (Liu et al., 2013, 2022; Garnham et al., 2015). These CTTs, which typically span approximately 17–24 amino acids, serve as the primary biochemical canvas for post-translational modifications (Liu et al., 2022; Nair et al., 2026).

These modifications do not act in isolation; instead, they alter the biophysical properties of the microtubule lattice and dynamically regulate the binding affinity of microtubule-associated proteins (MAPs) and molecular motors, such as kinesins and dynein (Praveen and Talwar, 2025; Teoh and Bartolini, 2025; Nair et al., 2026).

The code also operates through hierarchical and competitive interactions among PTM enzymes. Tubulin glycylation is initiated by TTLL-family glycylases, including TTLL3, and glycylation can compete with glutamylation for overlapping residues on the beta-tubulin C-terminal tail (Wloga et al., 2009; Garnham et al., 2017). Importantly, the elongating polyglycylase mechanism is species-dependent: the human TTLL10 protein has lost detectable elongating glycylase activity, suggesting that human tubulin glycylation may rely predominantly on monoglycylation rather than long polyglycine chains (Rogowski et al., 2009). This illustrates the broader principle that the tubulin code is combinatorial but also species- and cell-type-specific.

Genetic perturbations of these isotypes underscore their structural indispensability in neurovascular development and vascular pathology (Nair et al., 2026). Deletion or mutation of TUBA1A, TUBB2B, or TUBB3 in rodents and humans results in severe malformations of cortical development (MCD), including cortical dysplasia, abnormal neuronal migration, cerebral dysplasia, microdysgenesis, and clinical manifestations such as cognitive impairment, motor dysfunction, and epileptic seizures (Nair et al., 2026).

Additionally, mutations in these tubulin isotypes disrupt blood vessel morphology, impair proplatelet formation, and alter megakaryocyte growth, demonstrating that tubulin integrity is equally vital for maintaining the structural and functional homeostasis of the neurovasculature (Nair et al., 2026). The principal neuronal tubulin isotypes and their relevance to post-stroke pathophysiology are summarized in Table 1.

**Table 1:** Tubulin isotypes relevant to neuronal architecture and post-stroke pathophysiology.

Tubulin isotype	Primary gene	Cellular/Tissue expression	Post-stroke pathophysiological relevance	Associated mutations and pathology	Reference
βIII-tubulin	TUBB3	Predominantly restricted to neurons	Possesses a unique C-terminal tail that confers resistance to oxidative stress	Mutations cause disorders of axon guidance	Nair et al., 2026
βII-tubulin	TUBB2A/TUBB2B	Highly expressed in adult neurons and cortical networks	Contributes to synaptic architecture and dendritic-spine maintenance	Mutations lead to malformations of cortical development (MCD)	Nair et al., 2026; Yang et al., 2023
α-tubulin	TUBA1A	Expressed during active stages of cortical development	Regulates radial glial progenitor differentiation and neuronal migration	Mutations cause cortical dysplasia and microdysgenesis	Nair et al., 2026; Moutin et al., 2021
β-tubulin	TUBB5	Ubiquitously expressed; highly active in cortical progenitors	Maintains structural integrity of progenitor cells and vascular endothelium	Mutations cause severe brain development defects	Nair et al., 2026
βIVa-tubulin	TUBB4A	Expressed in CNS neurons and oligodendrocytes; strong relevance to white-matter and oligodendroglial biology	Can alter whole-lattice stability and secondarily increase acetylated and detyrosinated tubulin; relevant to white-matter repair	Mutations cause H-ABC/hypomyelination and demyelination with mutation-specific neuronal and oligodendroglial defects	Duncan et al., 2017; Curiel et al., 2017; Jaramillo-Restrepo et al., 2026
α4A-tubulin	TUBA4A	Expressed abundantly in the adult central nervous system	Features the longest polyglutamyl side chains; scaffolds key kinases and Tau	Dysregulation promotes Tau hyper-phosphorylation and microglia activation	Hausrat et al., 2022

### Beta-tubulin isotypes, whole-lattice dynamics, and oligodendroglial microtubules

Although detyrosination is a modification of the alpha-tubulin C-terminal tail, the functional substrate is an alpha/beta-tubulin heterodimer assembled into the microtubule lattice. Beta-tubulin isotype composition can therefore influence lattice conformation, dynamic instability, and the physical context in which alpha-tubulin PTMs are written and read. This principle is illustrated by TUBB4A-related tubulinopathy. In the taiep rat carrying the Tubb4a A302T mutation, Jaramillo-Restrepo et al. (2026) observed increased acetylated and detyrosinated tubulin in white-matter regions and increased acetylated tubulin in oligodendrocyte-marker-positive cells, with molecular modeling supporting a mutation-associated shift toward greater microtubule stability. Earlier studies similarly showed TUBB4A-associated microtubule accumulation, hypomyelination/demyelination, and mutation-specific neuronal and oligodendrocytic defects (Duncan et al., 2017; Curiel et al., 2017). These observations are relevant to stroke because they demonstrate that a beta-tubulin lesion can secondarily reshape the PTM state and mechanical behavior of the entire lattice and because post-ischemic white-matter repair depends on oligodendrocyte integrity as well as neuronal microtubules. Importantly, TUBB4A (beta-tubulin class IVa) is distinct from TUBA4A (alpha-tubulin 4A), which is discussed below in relation to polyglutamylation, GSK3beta, and Tau.

## The HDAC6-tubulin acetylation axis in cortical ischemia and synaptic collapse

The HDAC6-centered injury and rescue pathways discussed in this section are summarized schematically in Figure 2 and tabulated in Table 2.

**Figure 2:**
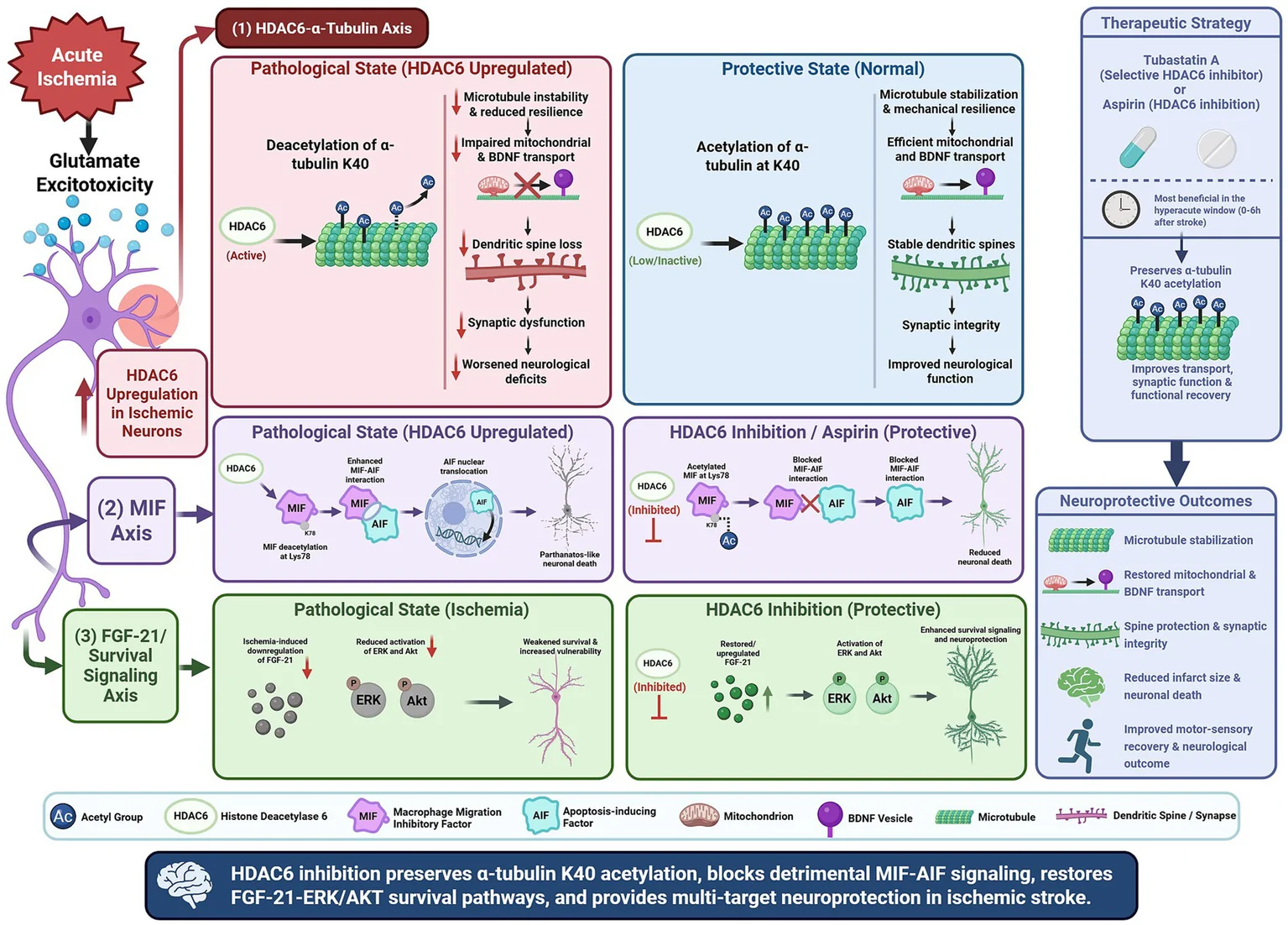
The HDAC6-alpha-tubulin acetylation axis and associated neuroprotective pathways in ischemic stroke. The schematic contrasts HDAC6-upregulated injury states with protective acetylation-preserving states and includes the MIF-AIF and FGF-21-ERK/Akt survival pathways. Created in BioRender. Hausburg (2026b). https://BioRender.com/jsvdt9l.

**Table 2:** HDAC6-related substrates and consequences of selective inhibition in ischemic stroke models.

Target substrate	Modification site	Direct cellular consequence of deacetylation	Structural/Functional outcome of selective inhibition	Reference
α-tubulin	Lysine 40 (K40), intraluminal	Microtubule instability, mechanical fragility, and axonal transport failure	Restores mitochondrial and BDNF transport; protects dendritic spines	Fessler et al., 2013; Wang et al., 2016; Yang et al., 2023
MIF	Lysine 78 (K78)	Promotes binding of MIF to AIF and subsequent nuclear translocation	Suppresses MIF-AIF complex assembly, blocking parthanatos-like neuronal death	Hu et al., 2022
FGF-21 pathway	Indirect transcriptional/metabolic regulation	Downregulates metabolic and mitochondrial survival signaling	Reverses FGF-21 downregulation, activating ERK and AKT cascades	Wang et al., 2016

One of the most heavily investigated modifications in the context of cerebral ischemia is the reversible acetylation of α-tubulin at the intraluminal Lysine 40 (K40) residue (Santiago-Mujika et al., 2021; Teoh and Bartolini, 2025; Nair et al., 2026). Unlike most other modifications that occur on the C-terminal tails, α-tubulin K40 acetylation occurs within the microtubule lumen (Santiago-Mujika et al., 2021; Teoh and Bartolini, 2025; Nair et al., 2026).

The specific acetyltransferase αTAT1/MEC17 (alpha-tubulin N-acetyltransferase 1) enters the lumen via surface diffusion to acetylate assembled tubulin (Teoh and Bartolini, 2025; Nair et al., 2026). Deacetylation is primarily executed by histone deacetylase 6 (HDAC6), a unique class IIb deacetylase that resides almost exclusively in the cytoplasm and targets non-histone substrates (Wang et al., 2016; Hu et al., 2022; Shi et al., 2022).

### Intraluminal K40 acetylation and excitotoxic transport failure

Under physiological conditions, α-tubulin K40 acetylation acts as a molecular “shock absorber,” enhancing the mechanical flexibility of microtubules, protecting them from mechanical breakage under strain, and facilitating retrograde and anterograde axonal transport of vital organelles like mitochondria and neurotrophins such as BDNF (Fessler et al., 2013; Wang et al., 2016; Teoh and Bartolini, 2025). During acute ischemic stroke, this homeostatic balance is severely disrupted (Wang et al., 2016; Hu et al., 2022). Experimental models of focal cerebral ischemia, such as middle cerebral artery occlusion (MCAO), demonstrate a robust, time-dependent upregulation of HDAC6 expression and enzymatic activity within the ischemic penumbra (Wang et al., 2016; Hu et al., 2022; Ma et al., 2026). Both HDAC6 mRNA and protein levels increase significantly at 3, 5, and 7 days post-ischemia, particularly in cortical neurons (Hu et al., 2022).

This pathological increase in HDAC6 activity leads to a dramatic reduction in α-tubulin acetylation levels in both the cortex and the striatum (Wang et al., 2016). The loss of tubulin acetylation destabilizes the microtubule network, causing microtubule depolymerization, dismantling axonal transport machinery, and impairing the delivery of mitochondria to energy-depleted synapses (Wang et al., 2016; Prota et al., 2023). This axonal transport deficit is replicated *in vitro* by treating primary cortical neurons with glutamate to mimic ischemia-induced excitotoxicity (Wang et al., 2016). Glutamate exposure causes the degeneration of over half of the cortical neurons and severely impairs mitochondrial motility within remaining neurites (Wang et al., 2016).

Pharmacological inhibition of HDAC6 using the highly selective inhibitor Tubastatin A (TubA) markedly restores the levels of acetylated α-tubulin, preserves microtubule stability, and improves motor-sensory functional deficits (Wang et al., 2016; Hu et al., 2022). Notably, Tubastatin A exhibits a prolonged therapeutic window of at least 24 h post-ischemia, which is significantly longer than that of pan-HDAC inhibitors like valproate, which are further limited by severe adverse effects on homeostatic cell cycle, inflammatory, and mitochondrial pathways (Wang et al., 2016; Ma et al., 2026).

Tubastatin A achieves this selective rescue with no significant effect on overall histone acetylation levels, confirming its highly specific action on the cytoplasmic HDAC6 isoform under ischemic conditions (Wang et al., 2016).

### MEC17 and post-stroke synaptic plasticity

Beyond axonal transport, α-tubulin acetylation is a critical determinant of dendritic spine structural integrity and postsynaptic density organization (Yang et al., 2023). Super-resolution imaging has shown that acetylated α-tubulin colocalizes directly with postsynaptic density 95 (PSD-95) in mature dendritic spines (Yang et al., 2023). Following cerebral ischemia, the rapid loss of acetylated α-tubulin correlates with the widespread collapse and degeneration of dendritic spines in the peri-infarct penumbra, directly contributing to the disruption of neural circuits (Yang et al., 2023).

Mice lacking αTAT1/MEC17 in cortical pyramidal neurons (Mec17 conditional knockout) do not exhibit changes in baseline spine density, but they suffer from significantly aggravated dendritic spine loss, severe synaptic injury, and profound motor deficits after ischemic stroke (Yang et al., 2023). Conversely, overexpressing αTAT1/MEC17 via adeno-associated virus (AAV) or administering Tubastatin A to preserve or elevate α-tubulin acetylation levels rescues PSD-95-positive mature dendritic spine density and promotes functional recovery (Yang et al., 2023).

### The MIF-AIF parthanatos pathway and metabolic reversal via FGF-21

While the recovery of microtubule-based transport is a major factor in neuroprotection, the therapeutic benefits of HDAC6 inhibition in stroke are also driven by non-tubulin deacetylation pathways. In a photothrombotic-stroke study, Hu et al. (2022) demonstrated that, under ischemic conditions, upregulated HDAC6 deacetylates Macrophage Migration Inhibitory Factor (MIF) at Lysine 78 (K78).

Deacetylated MIF exhibits an enhanced binding affinity for Apoptosis-Inducing Factor (AIF), facilitating the recruitment and subsequent nuclear translocation of the MIF-AIF complex, which triggers parthanatos-like neuronal cell death (Hu et al., 2022).

Genetic ablation or pharmacological inhibition of HDAC6 (or treatment with aspirin, which promotes MIF acetylation) increases MIF acetylation (Hu et al., 2022). This site-specific modification sterically hinders the interaction between MIF and AIF, blocking nuclear translocation of the complex and rendering cortical neurons resilient to ischemia-induced cell death (Hu et al., 2022). The structural necessity of this site was demonstrated using MIF-K78R mutants, which completely reversed the neuroprotective effects of both HDAC6 inhibition and aspirin treatment in response to ischemic stroke (Hu et al., 2022).

As such, wild-type mice exhibit severe brain infarction on Day 3, with an average increased infarct volume of the cortex (Hu et al., 2022). In contrast, MIF-K78Q acetylation-mimetic mutant mice exhibit a significantly reduced cortical infarct volume, which correlates with superior sensory-motor outcomes across multiple behavioral metrics, including the modified neurological severity score (mNSS), adhesive-removal test, and foot fault test (Hu et al., 2022).

Additionally, HDAC6 inhibition targets downstream metabolic signaling pathways (Wang et al., 2016). Cerebral ischemia induces a profound downregulation of fibroblast growth factor-21 (FGF-21), a circulating metabolic regulator of glucose and lipid metabolism induced by metabolic stress (Wang et al., 2016).

Treatment with Tubastatin A (TubA) significantly reverses this post-ischemic downregulation of FGF-21 in both the cortex and the striatum on Days 1 and 3 post-stroke, promoting cell survival by activating the downstream extracellular signal-regulated kinase (ERK) and AKT signaling pathways within the ischemic hemisphere (Wang et al., 2016). These convergent pathways support a model in which selective, early HDAC6 inhibition can preserve transport-competent microtubules while suppressing non-tubulin death signaling, as shown in Figure 2.

## The detyrosination cycle, delta-2 accumulation, and the Vasohibin-2 survival axis

The detyrosination-tyrosination cycle represents a highly dynamic PTM system that operates on the extreme C-terminal tail of α-tubulin (Nair et al., 2026). Newly synthesized and dynamic microtubules are typically tyrosinated (Garnham et al., 2015; Nair et al., 2026). Once polymerized and stabilized, the terminal tyrosine residue is enzymatically cleaved by tubulin carboxypeptidases—principally the Vasohibin-1 (VASH1) and Vasohibin-2 (VASH2) enzymes, which require heterodimerization with Small Vasohibin-Binding Protein (SVBP) for catalytic stability and protection from ubiquitin-mediated degradation (Moutin et al., 2021; Nair et al., 2026; Wei et al., 2026).

This detyrosination mark is subsequently read by molecular motors; for instance, kinesin-1 and kinesin-2 exhibit a high affinity for detyrosinated tracks, whereas cytoplasmic dynein preferentially binds to tyrosinated microtubules to initiate retrograde transport (Teoh and Bartolini, 2025; Nair et al., 2026). This cycle is completed when soluble, detyrosinated tubulin dimers are re-tyrosinated by Tubulin Tyrosine Ligase (TTL) (Moutin et al., 2021; Teoh and Bartolini, 2025; Nair et al., 2026).

### Eraser dynamics and mitophagy deficits

In both cardiomyocytes and neurons, the hyper-stabilization of microtubules via excessive detyrosination causes severe mitochondrial dysfunction and energetic collapse (Miura et al., 2025). High levels of detyrosinated α-tubulin directly suppress Parkin-mediated mitophagy, a critical quality control pathway that degrades damaged mitochondria (Miura et al., 2025).

This was demonstrated in TTL knockout (TTL-KO) and VASH2 overexpressing (VASH2-OE) cell lines, which accumulate high amounts of detyrosinated α-tubulin and exhibit reduced mitochondrial respiration, diminished mitochondrial membrane potential, and suppressed mitophagy—evidenced by decreased Mito-Keima fluorescence, reduced LC3 lipidation, and a marked depletion of ubiquitinated mitochondrial proteins (Miura et al., 2025).

During acute ischemic stroke, this mitochondrial clearance pathway is compromised. When neurons undergo hypoxia-induced energetic stress, the accumulation of damaged, ROS-producing mitochondria cannot be resolved due to detyrosination-mediated mitophagy blockade, driving the penumbral tissue toward progressive necrosis and apoptosis (Miura et al., 2025; Cui et al., 2026).

### The irreversible conversion to delta-2 tubulin

If detyrosinated microtubules persist without re-tyrosination, they undergo further enzymatic degradation (Moutin et al., 2021; Teoh and Bartolini, 2025). Cytosolic carboxypeptidases (specifically CCP1, CCP4, and CCP6) can cleave the penultimate glutamate residue from the C-terminal tail (Moutin et al., 2021; Teoh and Bartolini, 2025).

This irreversible reaction generates Δ2-tubulin, permanently removing the tubulin subunit from the protective and dynamic detyrosination-tyrosination cycle (Vu et al., 2017; Teoh and Bartolini, 2025). While Δ2-tubulin is a marker of hyperstable microtubules in mature neurons, its abnormal accumulation is strongly associated with pathological axonopathy, the loss of mitochondrial transport, and progressive neurodegeneration (Vu et al., 2017; Bae et al., 2021; Moutin et al., 2021; Teoh and Bartolini, 2025).

During cerebral ischemia, the loss of TTL activity accelerates the accumulation of detyrosinated and Δ2-tubulin, leading to structural stiffness and a breakdown in the sorting of polarized cargo within injured axons (Teoh and Bartolini, 2025). Depiction of the detyrosination-to-delta-2 axis is incorporated into Figure 3 together with polyglutamylation and lactylation pathways.

**Figure 3:**
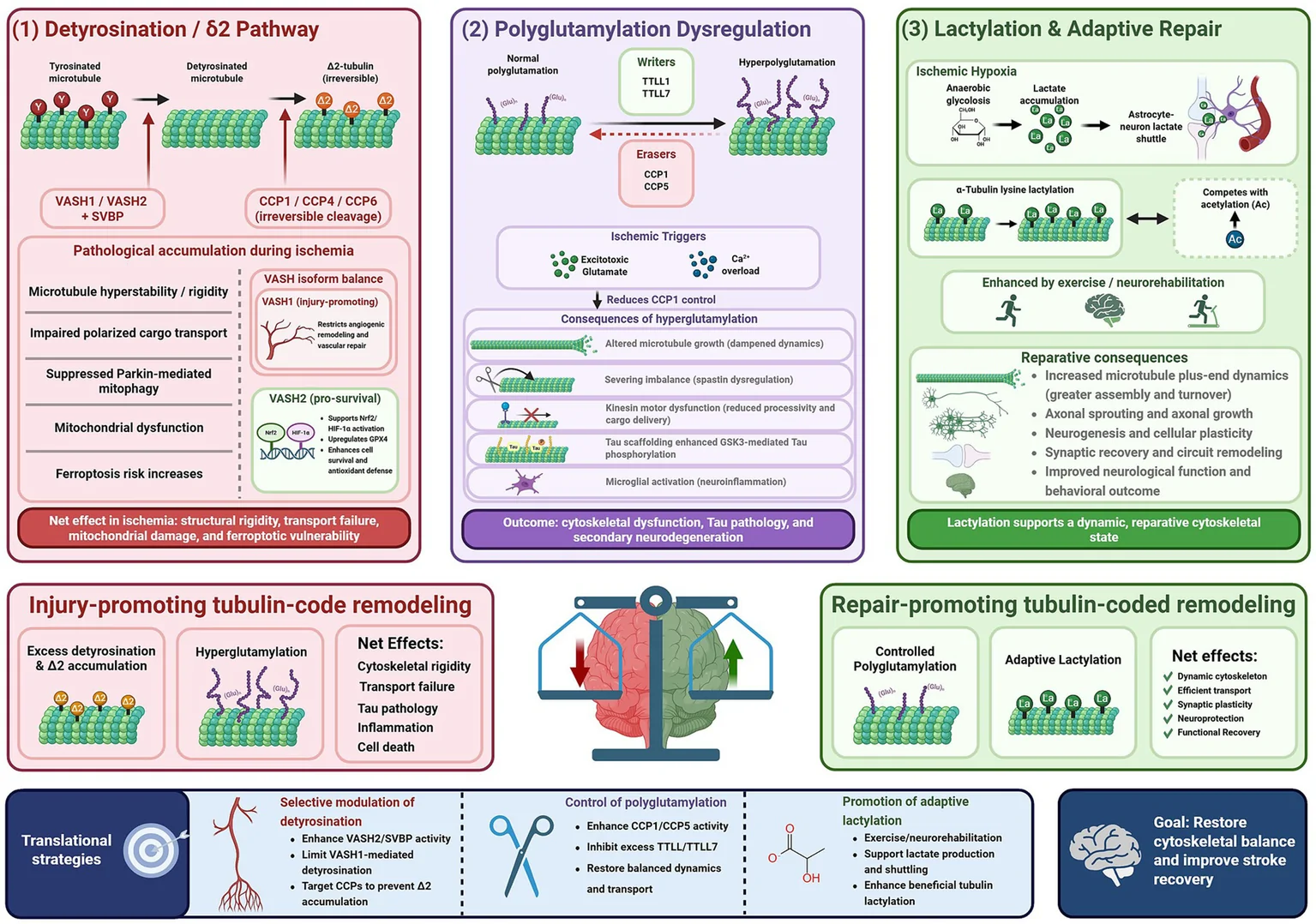
Detyrosination, polyglutamylation, and lactylation as tubulin-code determinants of injury and recovery after ischemic stroke. The schematic integrates VASH/CCP-dependent detyrosination, TTLL/CCP-dependent polyglutamylation, and lactate-associated alpha-tubulin lactylation as competing injury-promoting and repair-promoting programs. Created in BioRender. Hausburg (2026c). https://BioRender.com/jsvdt9l.

### The vasohibin isotype paradox and ferroptotic control

In the context of acute ischemic stroke, the detyrosination enzymes VASH1 and VASH2 exhibit highly paradoxical, divergent roles (Sun et al., 2025). Although both isoforms share high sequence homology and catalyze the same biochemical reaction, their genetic manipulation yields opposite phenotypes in mice subjected to transient MCAO (Sun et al., 2025).

VASH1 is actively transcribed in endothelial cells in response to VEGF via the VEGF-VEGFR2 axis, acting as an autocrine negative feedback inhibitor of angiogenesis that stabilizes nascent vessels during development but restricts vascular plasticity under stress (Nair et al., 2026; Wei et al., 2026). During the acute phase of stroke, VASH1 upregulation restricts penumbral vascular remodeling (Wei et al., 2026). Consequently, genetic reduction or targeted inhibition of VASH1 exhibits a clear neuroprotective effect, facilitating adaptive angiogenesis, vascular normalization, and improved perfusion in the peri-infarct tissue (Sun et al., 2025). Conversely, siRNA-mediated knockdown of VASH1 in endothelial cells under basal conditions can trigger premature cellular senescence—indicated by increased senescence-associated β-galactosidase (SA-β-gal) reactivity, flattened cell morphology, and the phosphorylation of p38 kinase—which enhances stress-induced cell death and presents a potential safety hurdle for chronic, non-targeted therapies (Miyashita et al., 2012).

In contrast, VASH2 is crucial for maintaining cellular survival pathways during ischemic stress, and its loss of function is consistently detrimental across multiple readouts. Following transient MCAO, VASH2-deficient (Vash2−/−) mice exhibit significantly larger cerebral infarct volumes, worse motor recovery, and severe neurological deficits compared to wild-type and VASH1-deficient counterparts (Sun et al., 2025). This protective role is mediated by the regulation of transcription factor networks and programmed cell death pathways: in the ischemic brain, VASH2 expression supports the stability of Nuclear Factor Erythroid 2-Related Factor 2 (Nrf2) and Hypoxia-Inducible Factor 1-Alpha (HIF-1α) (Sun et al., 2025). When VASH2 is depleted, these crucial transcription factors are downregulated, resulting in the collapse of the cell’s antioxidant defenses and directly triggering ferroptosis—a non-apoptotic, iron-dependent form of programmed cell death characterized by intense lipid peroxidation (Sun et al., 2025). Consistent with this, in VASH2-deficient brains the expression of Glutathione Peroxidase 4 (GPX4), the master inhibitor of ferroptosis, is significantly downregulated, while the expression of Acyl-CoA Synthetase Long-Chain Family Member 4 (ACSL4), a key driver of ferroptosis, is highly elevated (Sun et al., 2025).

Thus, while broad-spectrum, non-selective small-molecule inhibitors of tubulin detyrosination (such as the epoxide-based covalent inhibitor epoY) are being developed to combat chronic neurodegeneration, their acute administration during ischemic stroke must be approached with caution due to the risk of exacerbating ferroptotic neuronal death via VASH2 inhibition (Sun et al., 2025).

### Structural stiffness across cardiac and neurological disease

The mechanical consequences of pathological detyrosination in cerebral ischemia share striking parallels with other cardiovascular diseases (Pietsch et al., 2024; Cui et al., 2026). In myocardial ischemia/reperfusion injury and heart failure with preserved ejection fraction (HFpEF), cardiomyocytes experience a marked upregulation of tubulin detyrosination and an over-stabilization of the microtubule network (Pietsch et al., 2024; Cui et al., 2026). This dense, detyrosinated network acts as a physical drag, increasing the passive stiffness of cardiomyocytes, impairing diastolic compliance, and disrupting the mechanical transmission of contractile forces (Pietsch et al., 2024; Cui et al., 2026).

In a hypertrophic cardiomyopathy (HCM) mouse model, Cui et al. (2026) showed that targeting this network by overexpressing TTL for 12 weeks reduces the abundance of detyrosinated microtubules, successfully restoring diastolic filling, compliance, stroke volume, and cardiac output.

Stroke is not unique in linking PTM imbalance to loss of useful microtubule dynamics. In Alzheimer disease, impaired re-tyrosination is associated with accumulation of acetylated tubulin and reduced microtubule dynamics (Martínez-Hernández et al., 2022). In hereditary spastic paraplegia, spastin depletion produces abnormally long, hyperglutamylated microtubules, reduces kinesin binding and processivity, and impairs synaptic cargo transport (Lopes et al., 2020). In Huntington disease, reduced tubulin acetylation has been linked to impaired BDNF vesicular transport, which can be improved experimentally by HDAC6 inhibition (Dompierre et al., 2007). TUBB4A-related H-ABC provides the complementary example of excessive microtubule stabilization in oligodendrocytes (Jaramillo-Restrepo et al., 2026). Together, these disorders argue against equating “more stable” with “more protective.” The relevant biological objective is an appropriate range of microtubule dynamics for the cell type and disease phase.

In the brain, a similar mechanical stiffening occurs (Cui et al., 2026). The accumulation of stable, detyrosinated and Δ2-tubulin in ischemic axons and astrocytes creates a rigid, non-compliant cytoskeletal barrier (Vu et al., 2017; Moutin et al., 2021; Cui et al., 2026). This structural rigidity impedes growth cone motility, suppresses dynamic microtubule exploration at the plus-ends, and prevents the synaptic remodeling necessary for functional rewiring (Santiago-Mujika et al., 2021). In the context of acute ischemic stroke, this loss of structural plasticity may directly constrain the peri-infarct axonal sprouting and synaptic reorganization required for post-stroke functional recovery.

## Excitotoxic downregulation of CCP1 and the failure of polyglutamylation control

Polyglutamylation is a prominent post-translational modification in the adult mammalian brain, where it progressively accumulates during postnatal neuronal maturation (Garnham et al., 2015; Valenstein and Roll-Mecak, 2016; Ping et al., 2023). This ATP-dependent process involves the covalent attachment of glutamate side chains—ranging from single residues to complex polymers of up to six glutamates—to specific internal glutamate residues on the C-terminal tails of both α- and β-tubulin (Liu et al., 2013; Garnham et al., 2015; Chen et al., 2023).

The initiation and elongation of these glutamate chains are catalyzed by specific enzymes of the tubulin tyrosine ligase-like (TTLL) family (Garnham et al., 2017; Chen et al., 2023; Ping et al., 2023). In the central nervous system, TTLL1 acts as the primary writer for α-tubulin polyglutamylation, whereas TTLL7 is responsible for modifying β-tubulin (Garnham et al., 2015; Ping et al., 2023). This modification is reversed by cytosolic carboxypeptidases (primarily CCP1 and CCP5), which act as erasers to trim or entirely remove these glutamate side chains (Ramadan et al., 2021; Chen et al., 2023).

In healthy neurons, polyglutamylation functions as a biophysical rheostat that regulates the binding of MAPs and the activity of microtubule-severing enzymes (Liu et al., 2013; Valenstein and Roll-Mecak, 2016; Teoh and Bartolini, 2025). Specifically, the AAA + ATPase spastin, which is responsible for severing microtubules to generate new seeds for polymerization, is regulated in a nonlinear, biphasic manner by the number of glutamate residues on the tubulin tail (Liu et al., 2013; Valenstein and Roll-Mecak, 2016).

Spastin activity increases as the number of glutamates per tubulin rises from one to eight, but is inhibited when the glutamylation threshold exceeds eight residues (Valenstein and Roll-Mecak, 2016). This tight regulation is critical; either hyperglutamylation or hypoglutamylation disrupts microtubule-dependent organelle transport and causes progressive neurodegeneration (Garnham et al., 2015; Ramadan et al., 2021; Chen et al., 2023).

Specifically, hyperglutamylation occurs during the acute phase of ischemic stroke, when excessive accumulation of glutamate in the synaptic cleft overstimulates NMDA receptors, triggering a massive influx of intracellular calcium (Ca^2+^) (Esmaili-Shahzade-Ali-Akbari et al., 2026). This calcium overload impairs mitochondrial membrane potential, increases reactive oxygen species (ROS) production, and activates destructive calpain pathways (Liu et al., 2022; Esmaili-Shahzade-Ali-Akbari et al., 2026).

Crucially, this excitotoxic cascade directly targets the polyglutamylation eraser machinery (Ramadan et al., 2021). Under healthy conditions, CCP1 expression is highly active, preventing the pathological hyperglutamylation of microtubules (Ramadan et al., 2021). However, glutamate-induced excitotoxic injury triggers a rapid and severe downregulation of CCP1 expression in injured neurons (Ramadan et al., 2021).

The loss of deglutamylase activity allows the uncontrolled accumulation of polyglutamyl chains on the microtubule lattice (Ramadan et al., 2021). This hyperglutamylation alters the electrical properties of the tubulin tails, slowing the rate of microtubule growth, increasing microtubule catastrophe rates, and disrupting the motility of kinesin motors (Chen et al., 2023; Ping et al., 2023).

Furthermore, because polyglutamylation acts as an intracellular reservoir of glutamate in neurons, the degradation of hyperglutamylated microtubules under ischemic stress can release free glutamate, fueling a feed-forward cycle of excitotoxicity and neuronal death (Liu et al., 2013). Experimental knock-down of CCP1 severely exacerbates excitotoxic neuronal death, whereas preserving or restoring CCP1 expression prevents microtubule dysfunction and protects neurons against excitotoxic injury (Ramadan et al., 2021).

### Scaffolding tauopathy: the TUBA4A-GSK3β axis

Pathological hyperglutamylation also drives post-stroke secondary neurodegeneration by promoting Tau pathology (Hausrat et al., 2022). In healthy neurons, the microtubule-associated protein Tau binds to and stabilizes microtubules, facilitating axonal transport (Hausrat et al., 2022; Praveen and Talwar, 2025).

However, during ischemia, hyper-phosphorylated Tau dissociates from the microtubule lattice, forming toxic oligomeric aggregates that may lead to synaptic loss and eventual cognitive decline (Hausrat et al., 2022; Praveen and Talwar, 2025). This interface is directly regulated by polyglutamylation on the α-tubulin isotype TUBA4A (Hausrat et al., 2022).

In mature adult brains, TUBA4A expression increases significantly, and its C-terminal tail features the longest and most densely polyglutamylated side chains among all tubulin isotypes (Hausrat et al., 2022). This highly localized negative charge on TUBA4A tails acts as a scaffolding platform that recruits both Tau and glycogen synthase kinase 3β (GSK3β) to the microtubule surface (Hausrat et al., 2022).

Under ischemic conditions, this co-localization brings GSK3β into proximity with Tau, promoting GSK3β-mediated hyper-phosphorylation of Tau (Hausrat et al., 2022). Hyper-phosphorylated Tau dissociates from the microtubule, leading to cytoskeletal disintegration, the formation of neurofibrillary tangles, and the activation of neighboring microglia, which drives secondary neuroinflammation (Hausrat et al., 2022; Praveen and Talwar, 2025).

On the contrary, disrupting TUBA4A polyglutamylation—as demonstrated in knock-in mice carrying specific C-terminal mutations (TUBA4A-ΔC)—prevents the recruitment of GSK3β and Tau to the microtubule, significantly reducing phospho-Tau levels, preventing toxic Tau aggregation, and suppressing post-ischemic microglia activation (Hausrat et al., 2022).

## Evolutionary and physiological adaptation of glycylation, palmitoylation, and lactylation

The biochemical diversity of the tubulin code extends to other non-classical modifications, including glycylation, palmitoylation, and the recently discovered lactylation (Nair et al., 2026; Zhang, M., et al., 2026, Zhang, Y., et al., 2026). These non-classical marks provide important context for the broader injury-repair balance summarized in Figure 3.

### Microtubule glycylation and palmitoylation

Tubulin glycylation consists of glycine addition to glutamate residues within the C-terminal tails and is best established in axonemal and ciliary microtubules. TTLL3 functions as a chain-initiating tubulin glycylase, and glycylation can oppose glutamylation through competition for overlapping C-terminal-tail sites (Wloga et al., 2009; Garnham et al., 2017). The elongation step is not conserved uniformly across species; notably, human TTLL10 has lost detectable elongating glycylase activity (Rogowski et al., 2009). Direct evidence that altered tubulin glycylation is a major determinant of acute ischemic neuronal injury remains limited. We therefore include glycylation as an example of tubulin-code cross-talk rather than as an established stroke mechanism.

Palmitoylation, a reversible lipid modification, involves the covalent attachment of a 16-carbon palmitic acid to cysteine residues of tubulin, primarily Cysteine 376 (C376) on α-tubulin, catalyzed by palmitoyl-S-acyl-transferases (Praveen and Talwar, 2025; Nair et al., 2026). Palmitoylation is reversed by acyl-protein thioesterases, specifically α/β-hydrolase domain-containing 17A (ABHD17A) (Praveen and Talwar, 2025; Nair et al., 2026).

The primary function of tubulin palmitoylation is to enhance the membrane association of microtubules (Praveen and Talwar, 2025; Nair et al., 2026). This modification is critical for stabilizing the connection between the microtubule transport network and vesicle membranes (Praveen and Talwar, 2025; Nair et al., 2026).

During axonal transport, palmitoylation of adapter proteins facilitates the physical anchoring of cargo vesicles to molecular motors, ensuring efficient retrogradely and anterogradely directed transport of neurotransmitters and synaptic components (Praveen and Talwar, 2025). Following an ischemic insult, the disruption of palmitoylation enzymes causes the detachment of cargo vesicles from the microtubule tracks, leading to localized transport blocks, synaptic vesicle depletion, and a rapid decline in synaptic transmission (Praveen and Talwar, 2025; Nair et al., 2026).

## Tubulin lactylation: the metabolic-epigenetic repair bridge

In the brain, glucose metabolism is tightly coordinated by the astrocyte-neuron lactate shuttle (ANLS) (Wang et al., 2026). Under physiological conditions, glycolytically active astrocytes produce L-lactate, which is exported into the extracellular space via monocarboxylate transporters 1 and 4 (MCT1 and MCT4) and rapidly imported into oxidative neurons via MCT2 to serve as a primary energy substrate and signaling molecule (Wang et al., 2026).

During ischemic stroke, the sudden onset of hypoxia forces surviving glia and neurons to undergo rapid glycolytic reprogramming (Wang et al., 2026; Zhang, M., et al., 2026). This glycolytic surge, combined with severe tissue hypoxia, leads to a massive accumulation of intracellular lactate, providing an abundant substrate for protein lactylation (Wang et al., 2026; Zhang, M., et al., 2026).

Lactylation involves the covalent attachment of a glycolytically derived lactyl group to the ε-amino group of lysine residues on target proteins (Zhang, M., et al., 2026). The mechanistic basis for alpha-tubulin lactylation was established by Sun et al. (2024), who identified HDAC6 as an alpha-tubulin lactyltransferase using lactate and mapped lactylation to K40, with increased microtubule dynamics and neurite outgrowth. A subsequent peer-reviewed mouse stroke study showed that post-stroke aerobic exercise increased alpha-tubulin lactylation and was associated with improved neurological recovery, reduced neuroinflammation, enhanced neural-cell proliferation, and greater synaptic plasticity; exogenous lactate reproduced several of these effects (Zhang, Y., et al., 2026). Because K40 can be either acetylated or lactylated, the two marks are mutually exclusive at that residue. Importantly, K40 acetylation should not be described simply as lattice “rigidification”: it is enriched on long-lived microtubules and increases mechanical flexibility and resistance to repeated mechanical stress (Portran et al., 2017). By contrast, K40 lactylation has been reported to enhance microtubule dynamics. These observations support the possibility that the same tubulin residue participates in a phase-dependent switch between mechanically resilient transport tracks and more dynamic, growth-permissive microtubules. The lactylation pathway and its relationship to detyrosination and polyglutamylation are summarized in Figure 3.

## Translational Frontiers: pharmacological stabilization and clinical constraints

Candidate microtubule-directed therapeutic approaches and key translational constraints are summarized in Table 3.

**Table 3:** Candidate tubulin- and cytoskeleton-directed therapeutic strategies and translational limitations.

Therapeutic compound	Route	Molecular mechanism	Functional/Structural *in vivo* outcome	Primary clinical hurdles and safety warnings	Reference
Epothilone B (EpoB)	Intraperitoneal/subcutaneous	Taxane-site binder; promotes α-tubulin acetylation	Reduces fibrotic scarring by retaining basement-membrane fibronectin; promotes long-range brainstem projections	High risk of dose-limiting peripheral neuropathy; long CNS retention	Prota et al., 2023; Yang et al., 2018; Kugler et al., 2020; Griffin et al., 2023
Epothilone D (EpoD)	Intraperitoneal (precise dose not reported in source)	Taxane-site binder; stabilizes dynamic microtubules	Preserves CA1 subfield microtubules; reduces contralateral forelimb foot faults	Highly toxic at higher doses, causing severe neurodegeneration in the dentate gyrus	Kugler et al., 2020; Xiong et al., 2019
Tubastatin A (TubA)	Intraperitoneal	Selective cytoplasmic HDAC6 inhibitor	Restores α-tubulin K40 and MIF K78 acetylation; activates FGF-21 signaling	Administration beyond the 24-h/sub-acute phase may impair long-term functional recovery	Wang et al., 2016; Hu et al., 2022; Shi et al., 2022; Ma et al., 2026

The ultimate translational objective of deciphering the tubulin code in stroke is the development of pharmacological therapies capable of preserving or rebuilding the damaged neuronal cytoskeleton (Barman et al., 2019; Kugler et al., 2020). Because microtubule depolymerization and fragmentation are hallmarks of both hyperacute ischemic damage and chronic axonal retraction, targeting microtubule stability has emerged as a promising therapeutic strategy (Yang et al., 2018; Xiong et al., 2019; Kugler et al., 2020).

### Paclitaxel failure and cytosolic calcium toxicity

Early attempts to utilize classical microtubule-stabilizing agents, such as paclitaxel (Taxol), in ischemic models were highly unsuccessful (Rodríguez-Sinovas et al., 2015). While paclitaxel is a potent chemotherapeutic agent that binds to the taxane site of β-tubulin, its high-affinity stabilization of microtubules is so pronounced that it induces a mitotic block and triggers apoptotic cell death (Foland et al., 2005; Rodríguez-Sinovas et al., 2015; Prota et al., 2023). In isolated hearts submitted to simulated ischemia and reperfusion, pretreatment with paclitaxel failed to reduce myocardial infarct size (Rodríguez-Sinovas et al., 2015). This lack of protection was driven by a significantly greater increase in cytosolic calcium levels within paclitaxel-treated cells compared to controls, which promoted hypercontracture and cell death (Rodríguez-Sinovas et al., 2015). Additionally, paclitaxel administration caused a concentration-dependent decrease in heart rate, a reduction in left ventricular developed pressure (a decline in the heart’s peak systolic contractile pressure, reflecting impaired pump function), and a severe increase in coronary perfusion pressure, indicating adverse vascular reactivity (Rodríguez-Sinovas et al., 2015). These systemic toxicities, combined with paclitaxel’s poor blood–brain barrier permeability and tendency to induce peripheral neuropathy, restrict its utility for central nervous system indications (Prota et al., 2023; Bae et al., 2021).

### Low-dose epothilone stabilization and scar resolution

To overcome these limitations, research has focused on the epothilone family of microtubule-stabilizing agents, Epothilone B and Epothilone D (Yang et al., 2018; Xiong et al., 2019; Kugler et al., 2020). Epothilones are microtubule-stabilizing compounds, several of which have substantial CNS penetration in preclinical models. Ixabepilone, a semisynthetic epothilone B analog, is FDA-approved for metastatic breast cancer, but epothilone B (patupilone) and epothilone D are not approved for stroke or other neurologic indications (Ibrahim, 2021). Their use in stroke therefore remains preclinical.

Crucially, the degree of microtubule stabilization exerted by epothilones at low, sub-toxic doses is mild (Barman et al., 2019; Griffin et al., 2023). Rather than arresting cell division, low-dose epothilones prevent the formation of dystrophic “retraction bulbs” at the injured tips of axons (Griffin et al., 2023). By moderately stabilizing disorganized microtubules, epothilones coax these retraction bulbs back into highly active, physiological growth cones (Griffin et al., 2023).

When administered within a precise therapeutic window, systemic (intraperitoneal or subcutaneous) administration of Epothilone B or D has been shown to improve long-term functional recovery following focal cortical stroke (Kugler et al., 2020). This markedly exceeds the roughly 3- to 4.5-h intravenous thrombolysis window and the approximately 24-h mechanical thrombectomy window used clinically, reflecting the distinct goal of epothilone therapy—promoting delayed structural repair rather than acute reperfusion. Weekly systemic injections of Epothilone D (initiated 1 day post-stroke and continued for 3 weeks) significantly reduce the number of contralateral forelimb foot faults in grid-walking and cylinder tasks reflecting improved locomotor coordination (the forelimb foot-fault test quantifies missteps during grid-walking as a measure of skilled locomotor coordination, while the cylinder test assesses forelimb-use asymmetry during rearing as a measure of sensorimotor lateralization) (Kugler et al., 2020). In contrast, local infusion of Epothilone D directly into the stroke cavity using a hyaluronan/heparin gel failed to yield any behavioral recovery or stimulate axonal sprouting, highlighting the necessity of systemic distribution to target wide-ranging, inter-hemispheric cortical circuits (Kugler et al., 2020).

Systemic epothilone treatment promotes recovery through a dual-action mechanism (Kugler et al., 2020; Griffin et al., 2023). First, it stimulates the sprouting of novel, functional axonal projections from the peri-infarct motor cortex into adjacent premotor areas, as well as enhancing long-range motor projections descending into the brainstem (Kugler et al., 2020). Second, it directly targets the hostile microenvironment of the peri-infarct tissue (Kugler et al., 2020; Griffin et al., 2023). Epothilone administration restricts the polarization and migration of scar-forming fibroblasts into the injury zone, thereby reducing the deposition of inhibitory chondroitin sulfate proteoglycans (CSPGs) and fibrotic scarring (Kugler et al., 2020; Griffin et al., 2023).

Specifically, Epothilone B treatment decreases fibrotic scarring by retaining perivascular fibronectin within the vascular basement membrane distal to the lesion site (Griffin et al., 2023). This restricts the invasion of fibrotic cells into the parenchymal space, leading to a permissive microenvironment that allows regenerative axons to penetrate the lesion zone (Griffin et al., 2023).

Furthermore, combining low-dose epothilone therapy with physical rehabilitation training yields powerful synergistic effects, resulting in improvements in locomotor kinematics and gait parameters—such as limb coupling, support, and stride length—that exceed those achieved by either intervention alone (Griffin et al., 2023).

### Biphasic hippocampal vulnerability and dosing dynamics

Despite their therapeutic promise, epothilones exhibit a highly sensitive, biphasic dose–response profile in the central nervous system that must be carefully managed (Xiong et al., 2019). In global cerebral ischemia models, Wistar rats treated with a low dose of Epothilone D post-stroke display preserved hippocampal microtubule networks and reduced neuronal death in the CA1 subfield (Xiong et al., 2019).

However, escalating the dose completely abolishes these neuroprotective effects (Xiong et al., 2019). Instead, high-dose Epothilone D exacerbates delayed neuronal death and induces severe, independent structural damage to the granule neurons of the dentate gyrus (Xiong et al., 2019).

This localized toxicity occurs because the pyramidal cells in the CA1 subfield and the granule neurons in the dentate gyrus are equipped with dynamically different baseline microtubule networks and distinct tubulin isotype compositions (Xiong et al., 2019). Consequently, a dose that stabilizes injured CA1 microtubules can over-stabilize and freeze the highly dynamic, plastic microtubule systems of the dentate gyrus, impairing baseline neurogenesis and synaptic remodeling (Xiong et al., 2019).

## Clinical roadblocks: comorbidity, biological heterogeneity, and target engagement

As translational efforts advance, several key pathophysiological and methodological barriers must be addressed to successfully bridge the gap between preclinical rodent stroke models and clinical trial design (Ma et al., 2026).

Diabetes, aging, and sex materially influence stroke biology and treatment response, but direct evidence that these variables systematically reprogram the neuronal tubulin code remains limited. Their relevance to this review is therefore best considered as modification of the biological context in which tubulin-directed therapies are delivered rather than as established tubulin-code mechanisms. One direct vascular connection is the HDAC6/tubulin-acetylation axis. In endothelial models, the HDAC6 inhibitor tubacin increased eNOS expression and nitric oxide signaling; in diabetic db/db mice it improved endothelial function and was associated with attenuation of ischemic brain injury (Chen et al., 2019). This observation suggests that therapies intended to modify neuronal tubulin acetylation may also act on the neurovascular compartment, a potentially important source of both benefit and heterogeneity.

Future preclinical studies should therefore test tubulin-directed interventions in aged and diabetic animals of both sexes while measuring target engagement directly in relevant cell populations, including neurons, oligodendrocytes, and vascular endothelium. The persistent male bias in preclinical HDAC6 studies further argues for sex-balanced experimental design (Ma et al., 2026). Until such studies are available, diabetes- and sex-related differences in no-reflow or transcriptional stress responses should not be interpreted as evidence of a defined tubulin-code mechanism.

### Temporal constraints of cytoprotective targeting

Meta-analyses of preclinical HDAC6 inhibition strategies demonstrate a pooled standardized mean difference (SMD) in cerebral infarction size for treated animals compared to controls (Ma et al., 2026).

However, mathematical modeling reveals a strict temporal constraint: while administering HDAC6 inhibitors during the hyperacute phase of stroke yields significant neuroprotection and functional recovery, sub-acute (delayed) HDAC6 inhibition significantly impairs long-term functional recovery (Ma et al., 2026). This temporal switch occurs because the baseline deacetylation of non-histone substrates is required to support microenvironmental remodeling, vascular repair, and synaptic pruning during the chronic phase of stroke recovery (Lisek et al., 2025; Ma et al., 2026). Therefore, future clinical trials must strictly constrain therapeutic delivery to the hyperacute phase and validate these tubulin-targeted agents across rigorous, randomized controlled trial principles utilizing aged and diabetic cohorts of both sexes (Ma et al., 2026).

## Limitations of the evidence base

This review is narrative and hypothesis-generating rather than systematic. Most mechanistic evidence linking tubulin PTMs to ischemic injury or recovery derives from rodent stroke models, cultured neurons, or mechanistically informative non-stroke disease models; direct human stroke data on cell-type-specific tubulin PTMs remain sparse. Cross-disease comparisons are therefore used to test biological plausibility and identify candidate mechanisms, not to establish stroke causality. In addition, the effects of tubulin modifications are strongly dependent on timing, cell type, subcellular compartment, and baseline microtubule state. Therapeutic proposals in this review should consequently be viewed as testable frameworks that require validation in aged and comorbid models of both sexes and, ultimately, in human tissue, imaging, or biomarker studies that demonstrate target engagement.

## Conclusion

The tubulin code provides a coherent framework for understanding why ischemic stroke produces both immediate cytoskeletal failure and delayed impairment of neural repair. Loss of alpha-tubulin K40 acetylation, excessive detyrosination and delta-2 tubulin accumulation, dysregulated polyglutamylation, and maladaptive Tau scaffolding favor transport failure, mitochondrial dysfunction, ferroptotic vulnerability, and neuroinflammation. In contrast, adaptive lactylation and controlled microtubule remodeling may support axonal sprouting, synaptic recovery, and functional plasticity. A translational interpretation is therefore phase-specific: early interventions should preserve transport-competent microtubules and suppress death signaling, whereas later interventions should avoid excessive stabilization and instead promote dynamic repair.

An additional implication is that the relevant “tubulin code” is unlikely to be uniform across the ischemic brain. Neurons, oligodendrocytes, astroglia, microglia, and vascular cells have distinct microtubule demands and may respond differently to the same PTM-directed intervention. Future studies should therefore resolve cell-type-specific target engagement rather than infer benefit from bulk-tissue changes alone.

The figures and tables in this review organize these concepts into a practical structure for hypothesis generation and future preclinical study design.

## References

[ref1] Bae E. H. Greenwald M. K. Schwartz A. G. Smith J. R. Patel S. L. Chen Y. . (2021). Chemotherapy-induced peripheral neuropathy: mechanisms and therapeutic avenues. Neurotherapeutics 18, 2384–2396. doi: 10.1007/s13311-021-01142-2 PMC8804039 34676514

[ref2] Barman S. Das G. Mondal P. Pradhan K. Bhunia D. Khan J. . (2019). Power of tyrosine assembly in microtubule stabilization and neuroprotection fueled by phenol appendages. ACS Chem. Neurosci. 10, 1506–1516. doi: 10.1021/acschemneuro.8b00497 30565916

[ref3] Chen J. Roll-Mecak A. Walczak C. (2023). Glutamylation is a negative regulator of microtubule growth. Mol. Biol. Cell 34:ar70. doi: 10.1091/mbc.E23-01-0030 PMC10295482 37074962

[ref4] Chen J. Zhang J. Shaik N. F. Yi B. Wei X. Yang X.-F. . (2019). The histone deacetylase inhibitor tubacin mitigates endothelial dysfunction by up-regulating the expression of endothelial nitric oxide synthase. J. Biol. Chem. 294, 19565–19576. doi: 10.1074/jbc.RA119.011317, 31719145 PMC6926467

[ref5] Cui L. Guo L. Yang L. Zhang Y. Ma C. (2026). Microtubule dynamics and post-translational modifications in cardiovascular diseases: mechanisms and therapeutic implications. Front. Cardiovasc. Med. 13:1792687. doi: 10.3389/fcvm.2026.1792687, 41958459 PMC13056834

[ref6] Curiel J. Rodríguez Bey G. Takanohashi A. Bugiani M. Fu X. Wolf N. I. . (2017). TUBB4A mutations result in specific neuronal and oligodendrocytic defects that closely match clinically distinct phenotypes. Hum. Mol. Genet. 26, 4506–4518. doi: 10.1093/hmg/ddx338, 28973395 PMC7462055

[ref7] Dompierre J. P. Godin J. D. Charrin B. C. Cordelières F. P. King S. J. Humbert S. . (2007). Histone deacetylase 6 inhibition compensates for the transport deficit in Huntington disease by increasing tubulin acetylation. J. Neurosci. 27, 3571–3583. doi: 10.1523/JNEUROSCI.0037-07.2007, 17392473 PMC6672116

[ref8] Duncan I. D. Bugiani M. Radcliff A. B. Moran J. J. Lopez-Anido C. Duong P. . (2017). A mutation in the Tubb4a gene leads to microtubule accumulation with hypomyelination and demyelination. Ann. Neurol. 81, 690–702. doi: 10.1002/ana.24930, 28393430 PMC5495199

[ref9] Esmaili-Shahzade-Ali-Akbari P. Azami Tameh A. Parsaei H. Barati S. Vahidinia Z. (2026). Atorvastatin and ischemic stroke: mechanisms and therapeutic applications. J. Neuropathol. Exp. Neurol. 85, 815–830. doi: 10.1093/jnen/nlag009, 42133673

[ref10] Fessler E. B. Chibane F. L. Wang Z. Chuang D. M. (2013). Potential roles of HDAC inhibitors in mitigating ischemia-induced brain damage and facilitating endogenous regeneration and recovery. Curr. Pharm. Des. 19, 5105–5120. doi: 10.2174/1381612811319280009, 23448466 PMC6322545

[ref11] Foland T. B. Dentler W. L. Suprenant K. A. Gupta M. L. Himes R. H. (2005). Paclitaxel-induced microtubule stabilization causes mitotic block and apoptotic-like cell death in a paclitaxel-sensitive strain of *Saccharomyces cerevisiae*. Yeast 22, 971–978. doi: 10.1002/yea.1284, 16134117 PMC3008355

[ref12] Gadadhar S. Bodakuntla S. Natarajan K. Janke C. (2017). The tubulin code at a glance. J. Cell Sci. 130, 1347–1353. doi: 10.1242/jcs.199471, 28325758

[ref13] Garnham C. P. Vemu A. Wilson-Kubalek E. M. Yu I. Szyk A. Lander G. C. . (2015). Multivalent microtubule recognition by tubulin tyrosine ligase-like family glutamylases. Cell 161, 1112–1123. doi: 10.1016/j.cell.2015.04.003, 25959773 PMC4465277

[ref14] Garnham C. P. Yu I. Li Y. Roll-Mecak A. (2017). Crystal structure of tubulin tyrosine ligase-like 3 reveals essential architectural elements unique to tubulin monoglycylases. Proc. Natl. Acad. Sci. USA 114, 6545–6550. doi: 10.1073/pnas.1617286114, 28576883 PMC5488916

[ref15] Griffin J. M. Smith A. R. Thompson L. J. . (2023). Rehabilitation enhances epothilone-induced locomotor recovery after spinal cord injury. Brain Commun. 5:fcad005. doi: 10.1093/braincomms/fcad005 PMC9893225 36744011

[ref16] Guo Y. Li J. Liu X. Ding H. Zhang W. (2025). Potential therapeutic targets for ischemic stroke in pre-clinical studies: epigenetic-modifying enzymes DNMT/TET and HAT/HDAC. Front. Pharmacol. 16:1571276. doi: 10.3389/fphar.2025.1571276, 40356977 PMC12066669

[ref17] Hausburg M. (2026a). Pathophysiological reprogramming of the tubulin code in acute ischemic stroke. Created in BioRender. Available online at: https://BioRender.com/jsvdt9l (Accessed September 18, 2026).

[ref18] Hausburg M. (2026b). The HDAC6-alpha-tubulin acetylation axis and associated neuroprotective pathways in ischemic stroke. Created in BioRender. Available online at: https://BioRender.com/jsvdt9l (Accessed September 18, 2026).

[ref19] Hausburg M. (2026c). Detyrosination, polyglutamylation, and lactylation as tubulin-code determinants of injury and recovery after ischemic stroke. Created in BioRender. Available online at: https://BioRender.com/jsvdt9l (Accessed September 18, 2026).

[ref20] Hausrat T. J. Janiesch P. C. Breiden P. Lutz D. Hoffmeister-Ullerich S. Hermans-Borgmeyer I. . (2022). Disruption of tubulin-alpha4a polyglutamylation prevents aggregation of hyper-phosphorylated tau and microglia activation in mice. Nat. Commun. 13:4192. doi: 10.1038/s41467-022-31776-5 PMC9300677 35858909

[ref21] Hu J.-X. Ma W.-J. He L.-Y. Zhang C.-H. Zhang C. Wang Y. . (2022). Macrophage migration inhibitory factor (MIF) acetylation protects neurons from ischemic injury. Cell Death Dis. 13:466. doi: 10.1038/s41419-022-04918-2, 35585040 PMC9117661

[ref22] Ibrahim N. K. (2021). Ixabepilone: overview of effectiveness, safety, and tolerability in metastatic breast cancer. Front. Oncol. 11:617874. doi: 10.3389/fonc.2021.617874, 34295806 PMC8290913

[ref23] Janke C. Bulinski J. C. (2011). Post-translational regulation of the microtubule cytoskeleton: mechanisms and functions. Nat. Rev. Mol. Cell Biol. 12, 773–786. doi: 10.1038/nrm3227, 22086369

[ref24] Jaramillo-Restrepo C. Piazza V. Hernandez-Morales I. Lopez-Juarez A. Reséndiz-Nava L. F. Flores-García C. . (2026). H-ABC tubulinopathy exhibits a cytoskeletal defect associated with microtubule stability. Neurobiol. Dis. 224:107420. doi: 10.1016/j.nbd.2026.107420, 42044700

[ref25] Kugler C. Thielscher C. Tambe B. A. Schwarz M. K. Halle A. Bradke F. . (2020). Epothilones improve axonal growth and motor outcomes after stroke in the adult mammalian CNS. Cell Rep. Med. 1:100159. doi: 10.1016/j.xcrm.2020.100159 PMC7762779 33377130

[ref26] Lisek M. Bochenska N. Tomczak J. Duraj J. Boczek T. (2025). Epigenetic regulation in ischemic neuroprotection: the dual role of HDACs and HATs in neuroinflammation and recovery. Antioxidants 14:1015. doi: 10.3390/antiox14081015 PMC12382868 40867911

[ref27] Liu Y. Garnham C. P. Roll-Mecak A. Tanner M. E. (2013). Phosphinic acid-based inhibitors of tubulin polyglutamylases. Bioorg. Med. Chem. Lett. 23, 4408–4412. doi: 10.1016/j.bmcl.2013.05.069 PMC3725182 23777780

[ref28] Liu C. Chen Y. Xie Y. Xiang M. (2022). Tubulin post-translational modifications: potential therapeutic approaches to heart failure. Front. Cell Dev. Biol. 10:872058. doi: 10.3389/fcell.2022.872058 PMC9039000 35493101

[ref29] Lopes A. T. Hausrat T. J. Heisler F. F. Gromova K. V. Lombino F. L. Fischer T. . (2020). Spastin depletion increases tubulin polyglutamylation and impairs kinesin-mediated neuronal transport, leading to working and associative memory deficits. PLoS Biol. 18:e3000820. doi: 10.1371/journal.pbio.3000820, 32866173 PMC7485986

[ref30] Ma O. B. Noack T. C. Trollope A. F. Moxon J. V. . (2026). A systematic review and meta-analysis assessing the influence of histone deacetylase 6 inhibition on brain infarction and neurological function following acute ischaemic stroke in rodent models. J. Cereb. Blood Flow Metab. 46, 625–638. doi: 10.1177/0271678X251405674 PMC12815625 41549345

[ref31] Magiera M. M. Janke C. (2014). Post-translational modifications of tubulin. Curr. Biol. 24, R351–R354. doi: 10.1016/j.cub.2014.03.032, 24801181

[ref32] Martínez-Hernández J. Parato J. Sharma A. Soleilhac J.-M. Qu X. Tein E. . (2022). Crosstalk between acetylation and the tyrosination/detyrosination cycle of alpha-tubulin in Alzheimer disease. Front. Cell Dev. Biol. 10:926914. doi: 10.3389/fcell.2022.926914, 36092705 PMC9459041

[ref33] Miura S. Misaka T. Sekine T. Ogawara R. Ichimura S. Tomita Y. . (2025). Detyrosinated α-tubulin mediates mitochondrial dysfunction and diastolic impairment in heart failure with preserved ejection fraction. FEBS Lett. 599, 2474–2490. doi: 10.1002/1873-3468.70119 40878827

[ref34] Miyashita H. Watanabe T. Hayashi H. Suzuki Y. Nakamura T. Ito S. . (2012). Angiogenesis inhibitor Vasohibin-1 enhances stress resistance of endothelial cells via induction of SOD2 and SIRT1. PLoS One 7:e46459. doi: 10.1371/journal.pone.0046459, 23056314 PMC3466306

[ref35] Moutin M.-J. Bosc C. Peris L. Andrieux A. (2021). Tubulin post-translational modifications control neuronal development and functions. Dev. Neurobiol. 81, 253–272. doi: 10.1002/dneu.22774, 33325152 PMC8246997

[ref36] Nair A. R. Saroj N. Kunwar A. (2026). Emerging roles of tubulin isoforms and their post-translational modifications in microtubule-based transport and cellular functions. Biomolecules 16:81. doi: 10.3390/biom16010081, 41594623 PMC12838686

[ref37] Pietsch N. Chen C. Y. Kupsch S. Bacmeister L. Geertz B. Herrera-Rivero M. . (2024). Chronic activation of tubulin tyrosination improves heart function. Circ. Res. 135, 910–932. doi: 10.1161/CIRCRESAHA.124.324387 PMC11465905 39279670

[ref38] Ping Y. Ohata K. Kikushima K. Sakamoto T. Islam A. Xu L. . (2023). Tubulin polyglutamylation by TTLL1 and TTLL7 regulate glutamate concentration in the mice brain. Biomolecules 13:784. doi: 10.3390/biom13050784 PMC10216738 37238654

[ref39] Portran D. Schaedel L. Xu Z. Thery M. Nachury M. V. (2017). Tubulin acetylation protects long-lived microtubules against mechanical ageing. Nat. Cell Biol. 19, 391–398. doi: 10.1038/ncb3481, 28250419 PMC5376231

[ref40] Praveen B. S. Talwar P. (2025). Influence of palmitoylation in axonal transport mechanisms in neurodegenerative diseases. Front. Cell. Neurosci. 19:1613379. doi: 10.3389/fncel.2025.1613379 PMC12358401 40831763

[ref41] Prota A. E. Lucena-Agell D. Ma Y. Estevez-Gallego J. Li S. Bargsten K. . (2023). Structural insight into the stabilization of microtubules by taxanes. eLife 12:e84791. doi: 10.7554/eLife.84791 PMC10049219 36876916

[ref42] Ramadan Y. H. Gu A. Ross N. McEwan S. A. Barr M. M. Firestein B. L. . (2021). CCP1, a tubulin deglutamylase, increases survival of rodent spinal cord neurons following glutamate-induced excitotoxicity. eNeuro 8:ENEURO.0431-20.2021. doi: 10.1523/ENEURO.0431-20.2021, 33688040 PMC8021396

[ref43] Rodríguez-Sinovas A. Abad E. Sánchez J. A. Fernández-Sanz C. Inserte J. Ruiz-Meana M. . (2015). Microtubule stabilization with paclitaxel does not protect against infarction in isolated rat hearts. Exp. Physiol. 100, 23–34. doi: 10.1113/expphysiol.2014.082925 25557728

[ref44] Rogowski K. Juge F. van Dijk J. Wloga D. Strub J.-M. Levilliers N. . (2009). Evolutionary divergence of enzymatic mechanisms for posttranslational polyglycylation. Cell 137, 1076–1087. doi: 10.1016/j.cell.2009.05.020, 19524510

[ref45] Santiago-Mujika E. Luthi-Carter R. Giorgini F. Kalaria R. N. Mukaetova-Ladinska E. B. (2021). Tubulin and tubulin post-translational modifications in Alzheimer’s disease and vascular dementia. Front. Aging Neurosci. 13:730107. doi: 10.3389/fnagi.2021.730107 PMC8586541 34776926

[ref46] Shi L. Song Z. Li C. Deng F. Xia Y. Huang J. . (2022). HDAC6 inhibition alleviates ischemia- and cisplatin-induced acute kidney injury by promoting autophagy. Cells 11:3951. doi: 10.3390/cells11243951 PMC9776591 36552715

[ref47] Sun H. Hu X. Bian Z. Yu H. Bian Y. Ota-Elliott R. S. . (2025). Impact of endogenous vasohibin 1 and vasohibin 2 gene expression on ferroptosis in a mouse cerebral infarction model. Neuroscience 585, 170–180. doi: 10.1016/j.neuroscience.2025.08.061 40889590

[ref48] Sun S. Xu Z. He L. Shen Y. Yan Y. Lv X. . (2024). Metabolic regulation of cytoskeleton functions by HDAC6-catalyzed alpha-tubulin lactylation. Nat. Commun. 15, 170–180. doi: 10.1038/s41467-024-52729-0 PMC11437170 39333081

[ref49] Teoh J. Bartolini F. (2025). Emerging roles for tubulin PTMs in neuronal function and neurodegenerative disease. Curr. Opin. Neurobiol. 90:102971. doi: 10.1016/j.conb.2025.102971, 39862522 PMC11839326

[ref50] Valenstein M. L. Roll-Mecak A. (2016). Graded control of microtubule severing by tubulin glutamylation. Cell 164, 911–921. doi: 10.1016/j.cell.2016.01.019, 26875866 PMC6459029

[ref51] Vu H. T. Akatsu H. Hashizume Y. Setou M. Ikegami K. (2017). Increase in α-tubulin modifications in the neuronal processes of hippocampal neurons in both kainic acid-induced epileptic seizure and Alzheimer’s disease. Sci. Rep. 7:40205. doi: 10.1038/srep40205 PMC5220350 28067280

[ref52] Wang Z. Leng Y. Wang J. Liao H.-M. Bergman J. Leeds P. . (2016). Tubastatin a, an HDAC6 inhibitor, alleviates stroke-induced brain infarction and functional deficits. Sci. Rep. 6:19626. doi: 10.1038/srep19626 PMC4726180 26790818

[ref53] Wang H. Shao K. Zhou R. Xi J. Zhang W. Hao Y. . (2026). Lactylation at the crossroads of metabolism and epigenetics in neuroinflammation. Front. Immunol. 17:1784112. doi: 10.3389/fimmu.2026.1784112 PMC13082974 42004974

[ref54] Wei K. Li H. Huang H. Ban Y. Luo X. Zhang C. . (2026). From microtubule remodeling to clinical translation: the multifaceted roles of vasohibin-1 in disease modulation. Front. Immunol. 17:1759658. doi: 10.3389/fimmu.2026.1759658, 41737219 PMC12926144

[ref55] Wloga D. Webster D. M. Rogowski K. Bré M. H. Levilliers N. Jerka-Dziadosz M. . (2009). TTLL3 is a tubulin glycine ligase that regulates the assembly of cilia. Dev. Cell 16, 867–876. doi: 10.1016/j.devcel.2009.04.008, 19531357

[ref56] Xiong T.-Q. Chen L.-M. Gui Y. Jiang T. Tan B.-H. Li S.-L. . (2019). The effects of epothilone D on microtubule degradation and delayed neuronal death in the hippocampus following transient global ischemia. J. Chem. Neuroanat. 98, 17–26. doi: 10.1016/j.jchemneu.2019.03.002 30872184

[ref57] Yang Y. Zhang X. Ge H. Liu W. Sun E. Ma Y. . (2018). Epothilone B benefits nigrostriatal pathway recovery by promoting microtubule stabilization after intracerebral hemorrhage. J. Am. Heart Assoc. 7:e007626. doi: 10.1161/JAHA.117.007626 PMC5850167 29348323

[ref58] Yang C. Chen X. Zhang C. Lei X. Lu Y. Wang Y. . (2023). Acetylated α-tubulin alleviates injury to the dendritic spines after ischemic stroke in mice. CNS Neurosci. Ther. 29, 2327–2338. doi: 10.1111/cns.14184, 36965035 PMC10352872

[ref59] Zhang M. Li Y. Chen J. Xu Y. Zhao W. (2026). The enzymatic mechanisms of lactylation and its role in gynecological diseases: a comprehensive review. Front. Immunol. 17:1828022. doi: 10.3389/fimmu.2026.1828022 PMC13171315 42148060

[ref60] Zhang Y. Tang X. Li Z. Wu H. Wang Z. Zhu Y. . (2026). Aerobic exercise enhances alpha-tubulin lactylation and neurological recovery after ischemic stroke. Cell Biosci. 16:103. doi: 10.1186/s13578-026-01608-w PMC13531800 42321890

